# Global Infectious Disease Policy

**DOI:** 10.3201/eid0809.020314

**Published:** 2002-09

**Authors:** Eric D. Mintz

**Affiliations:** Centers for Disease Control and Prevention, Atlanta, Georgia, USA

At the International Conference on Emerging Infectious Diseases 2002, held in Atlanta, the Centers for Disease Control and Prevention (CDC) released a document entitled, “Protecting the Nation’s Health in an Era of Globalization: CDC’s Global Infectious Disease Strategy,” which describes plans for controlling infectious diseases worldwide. The document outlines global partnerships and measures for improving capacity for disease surveillance and outbreak response and for applying proven public health tools to the control of emerging infectious diseases over the next decade. In addition, the document calls for strengthening global initiatives for disease control, conducting applied research on diseases of international importance, and building public health training and capacity worldwide.

International Emerging Infections Programs in various parts of the world will support the activities outlined in the global strategy document. The International Emerging Infections Program (IEIP), Thailand, is the first site in the network of IEIPs proposed in the plan. Through the IEIP network, modeled after the U.S. Emerging Infections Program, specialists will work with local ministries of health to support laboratory-enhanced, population-based surveillance for infectious diseases. Data from this surveillance will allow ministries of health to prioritize diseases, evaluate targeted interventions, and support global efforts to prevent and control disease. IEIPs will train local scientists and CDC personnel, provide diagnostic and epidemiologic resources when outbreaks occur, and serve as platforms for regional infectious disease control activities.

In December 2001, IEIP Thailand and the Southeast Asia Regional Office of the World Health Organization hosted a training course on anthrax, attended by 64 participants from 16 countries. In 2002, IEIP initiated an investigation of an increase in reported leptospirosis cases in Thailand through a study of hospitalized patients with febrile illness. IEIP Thailand is planning studies of respiratory illness and encephalitis later this year; a second IEIP site will be launched soon.

